# Single-Port Laparoscopic Surgery for Adnexal Mass Removal During Pregnancy: The Initial Experience of a Single Institute

**DOI:** 10.3389/fmed.2021.800180

**Published:** 2022-02-15

**Authors:** Ling Han, Qi Wan, Yali Chen, Ai Zheng

**Affiliations:** ^1^Department of Obstetrics and Gynecology, West China Second Hospital, Sichuan University, Chengdu, China; ^2^Key Laboratory of Birth Defects and Related Diseases of Women and Children, Ministry of Education, Sichuan University, Chengdu, China

**Keywords:** single-port laparoscopy, adnexal mass, pregnancy, obstetric outcome, ovarian mass

## Abstract

**Objective:**

Single-port laparoscopy has become a feasible and safe approach for the management of benign adnexal masses during pregnancy. To our knowledge, there are few reports on the feasibility and safety of single-port laparoscopy for adnexal mass removal during pregnancy. Our study reports the use of single-port laparoscopy in adnexal mass removal during pregnancy in our hospital.

**Methods:**

We included 10 cases of single-port laparoscopic surgery for adnexal mass removal during pregnancy in the West China Second University Hospital between January 2017 and March 2020. Median values were found using SPSS20. When the *p*-value was <0.05, the median and interquartile range were used. All patients provided informed consent.

**Results:**

The following median values were recorded: surgical time, 112.50 min; blood loss, 25 ml; postoperative hospital stay, 3 days; postoperative pain [visual analog scale (VAS)] at 6 h, 3; and postoperative pain (VAS) at 24 h, 2. Our study reported no postoperative spontaneous abortions. There was one preterm birth.

**Conclusion:**

Single-port laparoscopy appears to be safe for both the mother and the fetus.

## Introduction

Conventional laparoscopy has been widely used as a gold standard surgical method for adnexal mass removal. It is associated with shorter hospital stays, less operative pain, and fewer intraoperative complications when compared with laparotomy. Between 1:500 and 1:635, women require non-obstetric surgery during pregnancy (1). The most common gynecological non-obstetric surgery is adnexal mass removal, with an incidence rate between 0.1 and 2.4% (2). Single-port laparoscopic surgery (SPLS) has become a feasible and safe approach for the management of benign adnexal masses when compared with conventional laparoscopy (3). With the development of surgical experience in laparoscopic technology, it has been used more in pregnant patients. However, to our knowledge, there are few reports on the feasibility and safety of SPLS used in adnexal mass removal during pregnancy. Given the lack of consensus on adnexal mass treatment during pregnancy, we aimed to compile evidence regarding the safety and efficacy of SPLS as a treatment. Our study reports the use of SPLS in adnexal mass removal during pregnancy in our hospital.

## Materials and Methods

### Subjects

We included 10 cases of SPLS for adnexal mass removal during pregnancy in the West China Second University Hospital between January 2017 and March 2020. The inclusion criteria were as follows: (1) adnexal torsion or rupture was suspected during surgery and (2) the adnexal mass was continually increasing in size during the second trimester of pregnancy and was > 6 cm in diameter.

### Operative Techniques

First, we made a 2–3 cm umbilical incision longitudinally. We then inserted the single-port wound retractor into the incision and the port cap was fixed to the wound retractor. The single-port cap contained a gas inlet and four access ports (Kangji Medical). The other laparoscopic instruments were the same as those conventionally used, such as 30°, 10 mm laparoscopes that are placed into the pelvic cavity through the 10 mm port. Then, a pneumoperitoneum was established using CO_2_ insufflation of up to 10–15 mmHg, and the abdominal pressure was maintained at around 12 mmHg during surgery. The entire surgical procedure of ovarian cystectomy and suturing of the ovarian tissue within the abdomen was carried out through the single-port. The ovarian tissue was sutured with 2–0 absorbable suture materials, and topical hemostats, such as oxidized cellulose and bipolar hemostat forceps, were used when necessary to reduce the risk of bleeding and to shorten the surgical time if suturing was difficult. Finally, the cyst was retrieved through the umbilical incision and placed in a bag before the umbilical incision was sutured.

**Figure 1:**
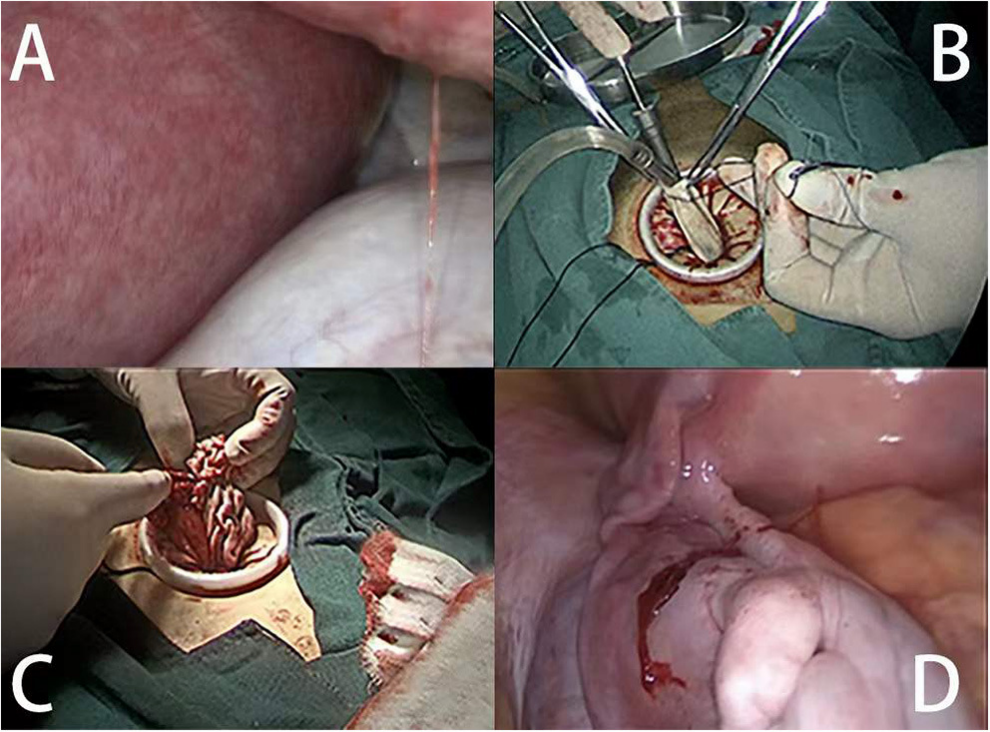
Extracorporeal cystectomy procedure. **(A)** The intraoperative view shows the enlarged uterus and left ovarian mass. **(B)** The puncture and aspiration of the contents of the ovarian mass. **(C)** Cystectomy was performed and sutured extracorporeally. **(D)** The ovarian tissue was returned to the abdomen.

Larger adnexal masses were removed using the single-port assisted extracorporeal method. In the single-port assisted extracorporeal method surgical procedure, after puncture and aspiration of the content of the ovarian mass, the cyst was extracted from the abdominal cavity through an umbilical incision. Ovarian cystectomy and suturing of the ovarian tissue were then performed outside of the abdomen (Figure 1).

### Data Collection and Statistical Analysis

Basic patient data was recorded. This included age, body mass index (BMI), surgical history, parity, method of conception, gestational week, maximum diameter of the ovarian mass, location of the ovarian tumors, and tumor pathology. The perioperative parameters included the diagnosis, such as the cause of the surgery, duration of the surgery, blood loss, postoperative hospital stay, axillary trocar insertion, intraoperative complications such as blood vessel injury, ileus injury, and postoperative complications such as ileus, fever, and wound infection. Postoperative pain was assessed at 6 and 24 h. All patients were contacted to determine the mode of delivery and the gestational week of delivery. Data on the duration of surgery, blood loss, postoperative hospital stay, postoperative pain [visual analog scale (VAS)] at 6 h, and postoperative pain (VAS) at 24 h were analyzed using SPSS20. When the *p*-value was < 0.05, the median and interquartile range were used.

## Results

A total of 10 cases of SPLS for adnexal mass removal during pregnancy were included in our study. Table 1 describes the basic characteristics of the included patients. Three of the ten procedures were performed because of torsion of the ovarian mass. The rest of the procedures were performed because of persistent enlarged ovarian masses which were >6 cm in the second trimester of pregnancy.

**Table 1:** Demographic characteristics of the patients.

**Case**	**Age**	**BMI**	**Number of previous surgeries**	**Maximum diameter of the ovarian cyst(cm)**	**Parity**	**Gestational week**	**Method of conception**	**Location of the ovarian mass**	**Postoperative diagnosis**
Case 1	25	20.3	0	10	G2P0+1	16 + 1	Natural	Left	Torsion of left ovarian mass
Case 2	26	21.6	0	8	G1P0	16 + 4	Natural	Right	Right ovarian mass
Case 3	31	25.7	0	6	G2P0+1	13 + 2	Natural	Right	Right ovarian mass
Case 4	24	18.4	0	10	G1P0	17 + 6	Natural	Bilateral	Bilateral ovarian mass
Case 5	33	21.5	0	11	G2P0+1	14	Natural	Left	Torsion of left ovarian mass
Case 6	32	24.2	0	10	G1P0	18	IVF-ET	Bilateral	Bilateral ovarian mass
Case 7	29	20.9	0	6	G1P0	8 + 2	Natural	Right	Right ovarian mass
Case 8	28	21.6	0	6	G1P0	18 + 2	Natural	Right	Torsion of right ovarian mass
Case 9	35	24.8	0	25	G1P0	13 + 2	Natural	Left	Left ovarian mass
Case 10	32	27.1	0	12	G3P1+1	15 + 5	Natural	Right	Right ovarian mass

Table 2 presents the perioperative data of the patients. The median surgical time was 112.50 min (interquartile range, 88.75, 185). The median blood loss value was 25 ml (interquartile range, 20, 57.5). The median postoperative hospital stay was 3 days (interquartile range, 3, 4). The median postoperative pain score (VAS) at 6 h was 3 (interquartile range, 2, 3). The median postoperative pain score (VAS) at 24 h was 2 (interquartile range, 1.75, 2). The final histological pathology is included in Table 2. Mature teratomas accounted for 40% of the included cases. Hemorrhagic corpus luteal cysts were found in 30% of patients. Mucinous cystadenomas accounted for 20% of cases and only one borderline ovarian serous papillary cystadenoma occurred in the study.

**Table 2:** Perioperative characteristics of the patients.

**Case**	**Histological pathology**	**Surgery time (min)**	**Surgical blood loss (ml)**	**Postoperative Hospitalstay (days)**	**Ancillary trocar insertion**	**Intraoperative complication**	**Postoperative complication**	**Postoperativepain (VAS) 6h**	**Postoperativepain (VAS) 24h**
Case 1	Hemorrhagic corpus luteal cyst	90	30	3	No	No	No	3	2
Case 2	Mature teratoma	100	20	4	No	No	No	1	1
Case 3	Borderline ovarian serous papillary cystadenoma	185	20	3	No	No	No	2	2
Case 4	Mature teratoma	200	80	3	One	No	No	3	2
Case 5	Mature teratoma	185	20	6	One	No	No	7	4
Case 6	Hemorrhagic corpus luteal cyst	120	100	3	No	No	Delayed wound healing	3	2
Case 7	Hemorrhagic corpus luteal cyst	105	30	4	No	No	No	2	2
Case 8	Mature teratoma	120	10	3	No	No	No	3	0
Case 9	Mucinous cystadenoma	83	50	3	No	No	No	3	2
Case 10	Mucinous cystadenoma	85	20	4	No	No	No	2	2

Table 3 reports the obstetric outcomes of the patients. Seven patients delivered after a full-term pregnancy. One patient delivered at 34 + 4 gestational weeks, four patients delivered naturally, and six had a cesarean section delivery.

**Table 3:** Obstetric outcome of the patients.

**Case**	**Gestational age at delivery**	**Delivery method**
Case 1	37 + 6	Natural labor
Case 2	40	Natural labor
Case 3	39 + 1	Cesarean delivery
Case 4	39	Natural labor
Case 5	41	Cesarean delivery
Case 6	34 + 4	Natural labor
Case 7	38 + 3	Cesarean delivery
Case 8	40 + 2	Cesarean delivery
Case 9	39	Cesarean delivery
Case 10	40 + 3	Cesarean delivery

## Discussion

In the past, the laparoscopic approach to treat adnexal masses in the second and third trimesters of pregnancy has been discouraged. The main concerns were the risk of uterine perforation when using a Veres needle, the impact of intraabdominal pressure and CO_2_ on the feto-maternal circulation, longer surgical times compared to laparotomy, and potential harm from the use of monopolar current. Based on these concerns, open surgical techniques have been preferred during pregnancy. Pearl et al. (4) commissioned the guideline for laparoscopy in pregnancy which recommends that (1) End-tidal CO2 (ETCO_2_) be used as a surrogate marker for maternal arterial CO_2_ monitoring, (2) CO_2_ insufflation be performed to 10–15 mmHg, and (3) that the abdominal operating pressure of 12 mmHg be observed to maintain feto-maternal perfusion and optimal utero-placental blood flow. Additionally, bipolar hemostasis has been reported to be safe to use in the course of laparoscopic surgery performed during pregnancy (5). Further studies have since reported maternal and fetal safety using a laparoscopic surgical approach in pregnant patients (5, 6). Laparoscopic surgery in pregnant women has also been associated with faster recovery, shorter hospital stays, and fewer wound infections compared with laparotomy (7). Single-port laparoscopy also has the advantages of conventional laparoscopy without the risk of Veres needle injury and with less postoperative incision pain, shorter hospital stays, and ease of specimen extraction through the umbilical incision (8).

We reported 10 cases of adnexal mass removal during pregnancy using SPLS and the obstetric outcomes for each patient were optimal. We have also summarized the literature about the single port approach during pregnancy in Table 4 (8–16). To our knowledge, only seven studies have reported the use of SPLS for adnexal mass removal during pregnancy (8–14). Jiang et al. (8) reported 15 cases of cystectomy during pregnancy with no instances of missed abortion or preterm birth. Lee et al. (9) reported 14 women with intrauterine pregnancies who underwent SPLS for adnexal disease during pregnancy with good obstetric outcomes. Takeda et al. (10) reported 29 cases of adnexal mass removal during pregnancy, four of which resulted in preterm birth. Scheib et al., Kim et al., Dursun et al., and Xiao et al. (11–14) detailed two, one, nine, and six case reports, respectively, where SPLS was performed on an adnexal mass during pregnancy, and the obstetric outcomes were also good.

**Table 4:** Published literature of single port laparoscopy during pregnancy.

**References**	**Country**	**Disease during pregnancy**	**Cases**	**Surgical complications**	**Obstetric outcome**
Jiang et al. (8)	China	Acute abdomen	26	None	1 abortion, 4 preterm births (did not mention the gestational age)
Lee et al. (9)	Korea	Adnexal surgery	14	None	1 preterm birth (24 + 5 week) and 1abortion
Takeda et al. (10)	Japan	Adnexal masses	29	None	4 preterm births (did not mention the gestational age)
Scheib et al. (11)	USA	Adnexal Masses	9	None	1 Preterm birth (36 weeks)
Kim et al. (12)	Korea	Ovarian mass	1	None	Not available
Dursun et al. (13)	Turkey	Adnexal mass	2	None	1 preterm birth (32 weeks)
Xiao et al. (14)	China	Gynecological disease	13	None	4 preterm births (35-36+2 weeks)
Koh et al. (15)	Korea	Acute appendicitis	2	None	Not available
Cho et al. (16)	Korea	Acute appendicitis	12	2 superficial surgical site infections and 1 post-operative ileus.	1 abortion

According to the American College of Obstetrics and Gynecology committee (17), performing non-urgent laparoscopic surgery in the second trimester is the best option for adnexal mass removal during pregnancy. In women with persistent masses in pregnancy, the reported malignancy rate is 3.6 to 6.8% and the rate of torsion of adnexal masses during pregnancy is 10% (2). Persistently growing ovarian masses >6 cm in diameter in the second trimester can be considered for removal *via* elective surgery in cases of emergencies and malignancies (17). Diagnostic laparoscopy in the management of adnexal masses during pregnancy is safe unless clinical severity warrants laparotomy or malignancy is strongly suspected (4). In our study, we performed three emergency surgeries and seven elective surgeries.

Chong et al. (18) reported using a single-port assisted extracorporeal approach for the removal of an ovarian cyst that measured > 8 cm in diameter on preoperative imaging. Kim et al. (12) first reported the safety of this method in adnexal mass during pregnancy. We adopted a single-port assisted extracorporeal approach for Case 9. The remaining patients underwent single-port laparoscopy. Ancillary trocar insertion was performed in two patients.

A meta-analysis reported by Liu et al. (19) showed that using laparoscopy for ovarian cyst removal is associated with better maternal and obstetric outcomes when compared with laparotomy. Three studies have reported on the safety and feasibility of single-port laparoscopy for adnexal masses when compared to conventional laparoscopy (3, 20, 21). Wang et al., Lee et al. (3, 20) compared the perioperative outcomes of single-port laparoscopy and conventional laparoscopy in adnexal mass removals and reported no difference in the median operation time, the median decreased level of hemoglobin from preoperative to postoperative day, or the median duration of postoperative hospital stay. Furthermore, Yim et al. (21) reported no difference in postoperative pain scores, operative time, perioperative complications, intraoperative blood loss, or duration of hospital stay between single-port laparoscopy and conventional laparoscopy in adnexal disease. However, only two reports have compared the use of single-port laparoscopy for ovarian mass removal during pregnancy with the use of conventional laparoscopy, but they both concluded that the techniques had comparable perioperative surgical and pregnancy outcomes (8, 10). We report a median surgical time of 112.50 min, a median blood loss of 25 ml, a median postoperative hospital stay of 3 days, a median postoperative pain (VAS) score of 3 at 6 h, and a median postoperative pain (VAS) score of 2 at 24 h. These results are similar to those detailed by Liu et al. (19) in their report on the perioperative data of laparoscopy used in adnexal masses during pregnancy. Our study reported no postoperative spontaneous abortions and one preterm birth. SPLS seems to be a safe alternative to conventional laparoscopy in treating patients with adnexal masses that require removal during pregnancy. However, there are still some shortcomings and challenges of SPLS compared to conventional laparoscopy during pregnancy. The ability to maneuver instruments in one port is limited and the enlarged uterus influences the view and operating space. As a result, the British Society for Gynecological Endoscopy (BSGE) recommends that laparoscopic surgery during pregnancy be performed by advanced laparoscopic surgeons with appropriate training and competencies (4).

There are two possible areas of future improvement for this technique. Minilaparoscopy uses 2–5 mm diameter laparoscopic instruments that can improve the cosmetic outcomes of surgery (22, 23). Minilaparoscopic single-site surgery is a new surgical procedure that combines the advantage of minilaparoscopic and single-site surgery. Casarin et al. (24) reported on this procedure to perform bilateral salpingo-oophorectomy. It is possible that this technique can be applied to the treatment of adnexal mass during pregnancy. In addition, it has been suggested that Endo Bags be used to extract suspected malignant masses to prevent spillage and the chance of spillage is rarely existed (25). In single-port laparoscopy, the cyst can be placed in a bag after cystectomy and retracted through the umbilical incision. However, if the cyst is ruptured during the cystectomy, the spillage cannot be avoided. In this case, the ovarian mass can be placed in the Endo Bag before the cystectomy is performed to reduce the spillage as suggested by Laganà et al. (26).

## Conclusion

The limitation of our study was the small number of patients included; therefore, we did not compare our findings with those of traditional laparoscopy performed in our hospital. We intend to include more cases and conduct large randomized trials with long-term follow-up in the future. However, based on the data we included, SPLS appears to be safe for the mother and fetus.

## Data Availability Statement

The original contributions presented in the study are included in the article/supplementary material, further inquiries can be directed to the corresponding authors.

## Ethics Statement

The study was approved by the Ethics Committee of the West China Second University Hospital and informed consent was taken from all the patients. The patients/participants provided their written informed consent to participate in this study.

## Author Contributions

LH and YC were responsible for the conception of the study and manuscript drafting. AZ, QW, and LH contributed to the revision and final approval of the manuscript. All authors contributed to the article and approved the submitted version.

## Conflict of Interest

The authors declare that the research was conducted in the absence of any commercial or financial relationships that could be construed as a potential conflict of interest.

## Publisher's Note

All claims expressed in this article are solely those of the authors and do not necessarily represent those of their affiliated organizations, or those of the publisher, the editors and the reviewers. Any product that may be evaluated in this article, or claim that may be made by its manufacturer, is not guaranteed or endorsed by the publisher.
